# Nerve growth factor modulates toll-like receptor (TLR) 4 and 9 expression in cultured primary VKC conjunctival epithelial cells

**Published:** 2009-10-13

**Authors:** Alessandra Micera, Barbara Stampachiacchiere, Eduardo Maria Normando, Alessandro Lambiase, Sergio Bonini, Stefano Bonini

**Affiliations:** 1 CIR, Laboratory of Ophthalmology, University Campus Bio-Medico, Rome, Italy; 2 IRCCS-G.B. Bietti Eye Foundation, Rome, Italy; 3 Second University of Naples and Institute of Neurobiology and Molecular Medicine, National Research Council, Rome, Italy

## Abstract

**Purpose:**

To investigate if nerve growth factor (NGF) might modulate toll-like receptor (TLR) 4 and 9 expression in primary cultures of vernal keratoconjunctivitis (VKC)-derived conjunctival epithelial cells (VKC-ECs).

**Methods:**

Primary cultures of ECs were established from VKC (n=7) and healthy-control (n=5) conjunctival specimens. Primary untouched and short-term NGF-exposed VKC-ECs were analyzed for molecular (relative real-time PCR) and biochemical (confocal and fluorescence-activated cell sorting analysis of TLR4 and TLR9 expression. Data were compared to their untreated as well as stimulated healthy-control counterparts. Conditioned media were analyzed for interferon (IFN)-γ, interleukin (IL)-4, IL-10, and IL-12 p40 secretion.

**Results:**

Primary untouched VKC-ECs showed TLR4 increase and TLR9 decrease compared to their healthy-control counterparts. NGF exposure resulted in a strong upregulation of TLR4 and a moderate upregulation of TLR9 in few passages VKC-ECs. Both TLR4 and TLR9 upregulation occurred in a dose-dependent fashion and were supported by biochemical analysis. NGF triggered a dose-response release of IL-10 in VKC-ECs conditioned media, an effect not detected for IL-4, IL-12 p40, and IFN-γ.

**Conclusions:**

Our data indicate that NGF is able to induce TLR4/TLR9 overexpression in VKC-ECs. These cells exhibited poor IL-4, IL-12 p40, and IFN-γ responses to NGF, while a significant IL-10 decreased secretion was detected. The different NGF-induced TLR response between VKC and healthy-control conjunctival ECs as well as the different cytokine response might reflect a different pattern of cell activation according to the state of VKC.

## Introduction

Vernal keratoconjunctivitis (VKC) is a childhood, chronic, allergic eye disease, with a multifactorial pathogenesis [1]. The immune reaction is characterized by T helper subtype 2 lymphocytes cells, eosinophils, mast cells, and fibroblast infiltration and/or activation, together with a complex network of soluble mediators, which can lead to corneal complications [1–3]. Cytokines, growth factors, neuropeptides and other soluble mediators are increased in tears and active VKC and involve conjunctival epithelial cells (ECs) and fibroblasts in the inflammatory reaction [4,5]. Among these factors, nerve growth factor (NGF) plays pleiotropic effects on ECs, fibroblast and immune cells [6–8]. NGF, trkA^NGFR^, and p75^NTR^ are widely expressed in the healthy ocular surface and significantly change under pathological states [9–11]. NGF is affected in VKC blood and tarsal conjunctiva, implying a NGF possibility to modulate ocular inflammation and epithelial activities [11,12].

Conjunctival ECs play a significant pro-inflammatory role in VKC by participating in the local immune reaction throughout the synthesis of cytokines known to promote inflammation and expression of molecules (intracellular adhesion molecule-1) able to recruit inflammatory cells [13]. Being the first line of defense, ECs express a class of transmembrane receptors named the toll-like receptors (TLRs) [13,14]. TLRs activate the innate (host) immune reaction, launch the adaptive immune response, and modulate the Th1/Th2 cell balance in several allergic/autoimmune disorders [15–17]. In the ocular surface, the widespread TLR distribution offers a quick and selective response to pathogens [18,19]. TLR expression/function is highly dynamic and tightly regulated in response to encountered bacterial stimuli [15]. TLR variation during bacterial/viral infections as well as allergic/autoimmune inflammation highlights a more complex functional mechanism [18]. We demonstrated a TLR4 transcript upregulation and a TLR9 transcript downregulation in VKC-inflamed conjunctival epithelium, suggesting a TLR contribution in the pathogenesis of the disease [20].

Some recent findings propose an NGF contribution in the innate and adaptive immune responses [21,22]. Herein, primary cultures of VKC conjunctival ECs were checked for TLR4/TLR9 expression and then exposed to exogenous NGF to evaluate TLR4/TLR9 changes at either molecular and biochemical levels as well as cytokine release (interferon [IFN]-γ, interleukin [IL]-4, IL-10, and IL-12 p40) in the conditioned media.

## Methods

### Tissue sampling and establishment of primary cultures

A total of 7 patients with active VKC (5 male/2 female, mean age 15.71±5.59) and 5 sex/age-matched healthy-control patients, who underwent minor surgery, were included in the study. Active VKC diagnosis was based on clinical presentation, complete ophthalmic examination and basal histology (eosinophils in conjunctival scraping). Patients had recurrent itching, redness, photophobia, tearing in early spring associated with mild to severe cobblestone-like appearance of the upper tarsal conjunctiva, mucous discharge, and epithelial keratopathy. A conjunctival biopsy was obtained from the upper tarsal conjunctiva of both patients and controls. A signed consent was obtained from each informed participant (parents/patients). All protocols adhered to the tenets of the Declaration of Helsinki and the ARVO Statement in Ophthalmic and Vision Research for research involving human subjects and were performed  according to the Intramural committement. Specimens were cut into several pieces, put as explants in collagen-coated 24-well plates and left to attach for 10 min, before adding serum-free media (dF12 containing 100 U/ml penicillin and 100 μg/ml streptomycin; 37 °C, 5% CO_2_ in air), to favor the migration of ECs [23–25]. Cells outgrowing from explants (after 1 week of culturing) were maintained for an additional 10 days (P_0_) and split/expanded (P_1-2_) according to a standardized enzymatic-harvest protocol (DispaseII from ICN, Milan, Italy) [25]. Conjunctival ECs were screened for the selective expression of the defining marker cytokeratin 19 (mouse anti-human CK19; 1/100, Dako Corp., Carpinteria, CA) and the absence of the fibroblast contaminants Thy-1/α-Smooth Muscle Actin for Fibroblasts (FB)/myofibroblasts (myoFBs; Dako). In case of mycoplasm contamination (Hoechst staining), the cells were one passage-treated with Mycoplasm Removal Agent (5 μg/ml; ICN). All molecular and biochemical reagents were purchased from ICN, SERVA (Weidelberg, Germany), and Euroclone (Milan, Italy), while sterile tissue culture plasticware were from Nunc (Roskilde, Denmark).

### NGF dose-response experiments

Few passage (P_1-2_) ECs were cultured for these studies in order to retain VKC-EC phenotype. The biological active murine 2.5S βNGF Grade I (herein referred to as NGF; Alomone Labs. Ltd, Jerusalem, Israel) was used for the specific dose-response experiments (1–250 ng/ml). Neutralizing anti-NGF antibodies were from R&D (Minneapolis, MN). After short-term exposure (24 h), monolayers were washed in Hanks’ Balanced Sodium Salt (HBSS) and directly used for confocal analysis or enzymatically harvested as single cells before performing molecular (relative real-time PCR) or biochemical (fluorescence-activated cell sorting [FACS]/ enzyme-linked immuno-sorbent assay [ELISA]) analysis. Conditioned media were assayed for cytokine expression. Cells from each specimen were used in independent experiments and not pooled.

### Confocal analysis

Monolayers were post fixed in 4% buffered-paraformaldehyde (PFA; prepared in 1× phosphate-buffered saline [PBS], pH 7.5) and quenched in 50 mM NH_4_CL before fluorescence staining. A brief blocking/permeabilizing step (0.8% bovine serum albumin [BSA] and 0.3% Triton-X100 in PBS) was performed before the addition of the specific antibodies: rabbit anti-human TLR4 (sc-10741; Santa Cruz Biotech, Santa Cruz, CA), goat anti-human TLR9 (sc-16247; Santa Cruz Biotech), mouse anti-human trkA^NGFR^ and goat anti-human p75^NTR^ antibodies (0.5–1 µg/ml; R&D). Specie-specific Cy2/Cy5-conjugated secondary antibodies (Jackson ImmunoResearch Europe Ltd, Suffolk, UK) were used to detect specific binding. The slides were closed using an anti-fading medium (Vectamount; Vector Laboratories, Inc., Burlingame, CA). Irrelevant isotype-matched IgG antibodies (Vector Laboratories, Inc.) were incubated in parallel and used as controls for the channel series acquisitions and related background subtraction. Oil immersion images were acquired by C1 software connected to an E4000 inverted microscope (Nikon, Tokyo, Japan). Figures were assembled in Adobe Photoshop 7.0 (Adobe Systems Inc., San Jose, CA) and minimal adjustments were made to the figure to obtain the best quality.

### Total RNA extraction, cDNA synthesis and relative real-time PCR

Total RNA was extracted by OMNIzol (Ambion, Milan, Italy) and run for real-time RT-PCR amplification (Opticon2 thermocycler; MJ Research, Watertown, MA) with the gene-specific primers for the human *TLR4*, *TLR9*, *trkA^NGFR^*, *p75^NTR^*, *GAPDH* and *H3* genes (MWG, Biotech, Ebersberg, Germany) [8,20,26]. RNA was screened according to the spectrophotometer (ND1000; λ_260/280 _>1.8; NanoDrop Technologies, Wilmington, DE) as well as agarose gel size-fractionation analysis. cDNA was synthesized from 3 µg total RNA using Moloney Murine Leukemia Virus (MMLV) reverse transcriptase (IMPROM; Promega, Milan, Italy). For amplification, SYBR Green PCR Master Mix (Applied Biosystems, Foster City, CA) was used, and 47 cycles were run with denaturation at 94 °C/30 s and extension at 75 °C/30 s with the specific annealing temperatures [8,20,26]. A 60 °C to 95 °C melting curve was recorded for each sample/gene. Samples tested negative for DNA contamination, as detected by amplification of total RNA instead of cDNA. Negative controls (without template) and positive controls were produced for each plate run. In pilot experiments, amplicons were purified (Wizard® SV Gel and PCR Clean-Up System; Promega) and checked for amplicon identity. Samples were amplified in triplicate, and from these replicates, averages were calculated and expressed as real cycle threshold (Ct; ΔCt = ΔCt_target_- ΔCt_reference_) and reported as normalized ΔCts±SD or as an expression ratio of a normalized target gene (fold changes in log2-expression ratio), according to the formula 2^(-ΔΔCt)^, where ΔΔCt = ΔCt_sample_- ΔCt_calibrator_ (REST 384–2006 software) [27].

### FACS analysis

Single cells were processed for membrane/cytoplasm staining (TLR4/TLR9 diluted 1/100; Santa Cruz), according to a standardized protocol, including fixation (3.7% PFA in Hank’s Balanced Salt Solution (HBSS) and blocking/permeabilization (3% BSA and 0.3% Triton-X100 in HBSS) treatments. The indirect-immunofluorescence staining was carried out with specific primary antibodies followed by FC/PerCPCy5-coupled, specific, secondary antibodies (Jackson ImmunoResearch) in HBSS containing 0.3% Triton-X100 and 0.01% NaN_2_. Control fluorescent staining (isotype-matched antibodies from eBiosciences, San Diego, CA) was run in parallel for each set of experiments. Cells were evaluated by FACSCalibur^TM^ using CellQuestPro^TM^ software (Becton-Dickinson, San Jose, CA). Logarithmic and linear signals were acquired/analyzed from 5000 gated cells/sample and presented using WinMDI 2.5 software (the free software WinMDI, The Scripps Research Institute, La Jolla, CA). A logical gate (back-gating on FL3-CK19) combining CK19^+^cells and their scatter properties were used for identifying the EC population. Instrument setting, compensation and calibration were performed using 3-color calibration beads (bdbioscience [BD Biosciences], San Jose, CA).

### Cytokine secretion analysis

Conditioned media were collected and clarified by centrifugation before predilution and sample loading in 96-well precoated plates. IFN-γ, IL-4, IL-10, and IL-12 p40 levels were assayed using the commercially available (ELISA) kits, according to the manufacturer’s instructions (Biosource International, Camarillo, CA). Absorbance was detected using an ELISA reader (Tecan Systems, Inc., San Jose, CA). Normalization for total protein was achieved between the different treatments by A280 spectrophotometric analysis (Nanodrop). Cytokine release is expressed in the text as percentage of release in comparison to untreated sister cells.

### Statistical analysis

Data are expressed as the mean±SD (in the text) or mean±SEM (in figures). Parametric ANOVA coupled with Tukey–Kramer post hoc analysis was used to detect significant difference (statistical package StatView II for PC; Abacus Concepts. Inc., San Jose, CA). A p≤0.05 was considered as statistically significant.

## Results

**Figure 1:**
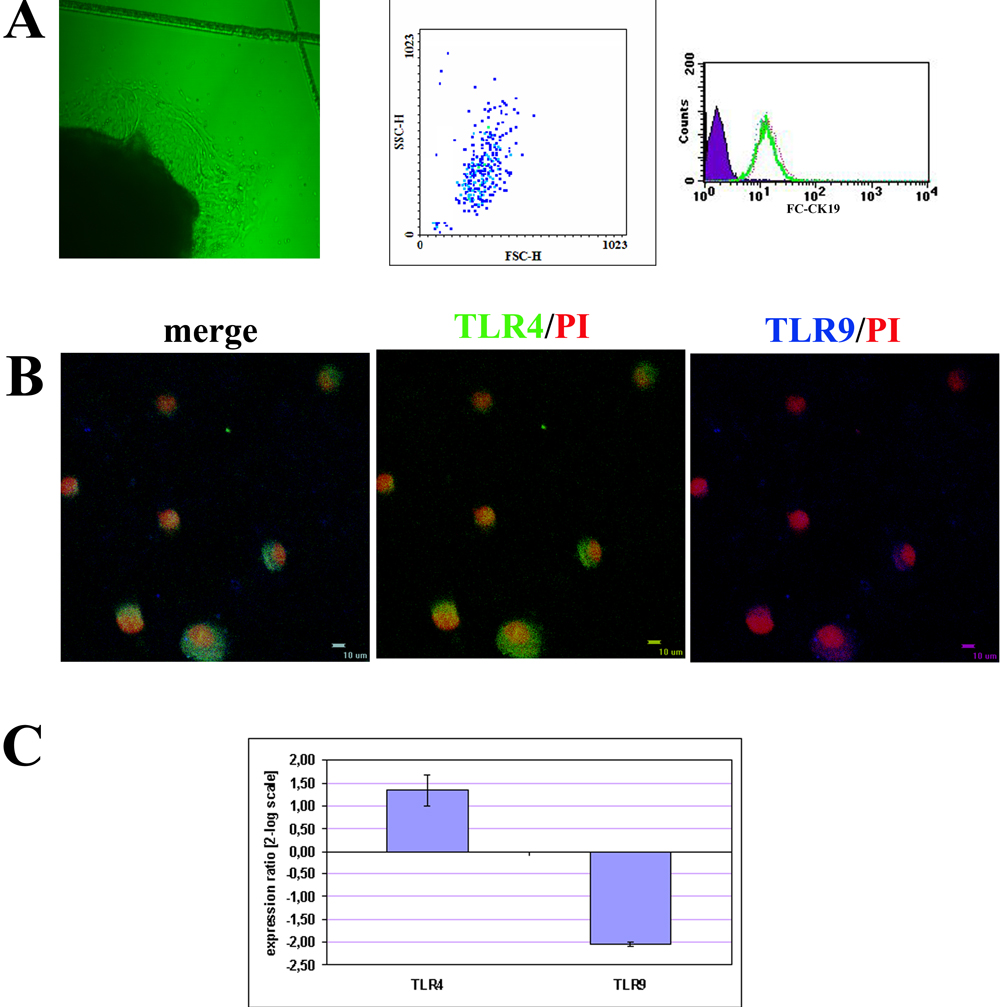
Characterization of primary conjunctival epithelial cells outgrew from VKC explants (VKC-ECs). **A**: Phase contrast microscopy (Normasky) showing VKC-ECs migrating from explants after 1 week (left, 10× optic field). The picture is representative of all VKC biopsies. A representative forward/side scatter plot of gated primary VKC-ECs (middle) and a FC-CK19 histogram demonstrating the purity of outgrew ECs (right; solid area: background staining (negative control); bold line: 97% CK19^+^ ECs). **B**: Confocal analysis specific for TLR4 and TLR9 (merge) in VKC-ECs. Control-isotype signal (data not shown) was used in channel series acquisitions, and identical acquisition settings were carried out for all images (60× oil immersion). **C**: *TLR4*/*TLR9* transcript expression in untouched primary cultures of VKC-ECs (P_0_; p<0.01, REST-ANOVA Turkey–Kramer-coupled analysis).

An increasing number of cuboidal cells with typical morphology of ECs migrated from the VKC conjunctival explants by 1 week of culturing (Figure 1A, left) and reached confluence by 10 days. At confluence outgrew cells were harvested and analyzed (P_0_) or replated for dose-response experiments (P_1–2_). As detected by FACS analysis, approximately 97% of gated VKC and healthy-control (P_0_) ECs stained positive for CK19, a defining marker of conjunctival ECs (Figure 1A, middle and right). Confocal analysis on cytospun of untouched primary (P_0_) VKC-ECs showed the presence of TLR4 (Cy2, middle) at either membrane or cytoplasm points, while TLR9 (cy5, Figure 1A, right) was mainly localized at the cytoplasm level (Figure 1B, merge). Particularly, a weak fluorescence for TLR9 was detected compared to those of TLR4. The in vitro studies were carried out on primary cultures (P_0_) and few passages (P_1-2_) conjunctival ECs in order to retain the in vivo VKC phenotype. As previously reported in humans, both trkA^NGFR^ and p75^NTR^ are expressed by VKC epithelium and primary cultures of ECs [10,12].

### Molecular expression of *TLR4* and *TLR9* in VKC conjunctival ECs

Spontaneous TLR4/TLR9 transcript expression was evaluated in these untouched primary (P_0_) VKC-ECs by relative real-time PCR. Briefly, in VKC-ECs ΔCt for *TLR4* was 9.85±5.34 and ΔCt for *TLR9* was 16.56±3.78, while in healthy-control ECs, ΔCt for *TLR4* was 10.16±2.57 and ΔCt for *TLR9* was 5.17±1.12, as detected after Ct normalization with reference genes (*GAPDH* and *H3*). Ct values are inversely proportional to gene expression. As shown in Figure 1C, the REST analysis of the single Ct values indicate that *TLR4* transcript was increased while *TLR9* transcript was decreased in primary VKC-ECs compared to their healthy-control counterpart (+1.23±2.71/-4.03±0.04 *TLR4*/*TLR9*, p<0.01 REST–ANOVA Tukey–Kramer-coupled analysis; ranging from -4.15±2.11/-2.37±0.26 *TLR4*/*TLR9* for mildly active to +1.77±5.90/-6.47±2.81 *TLR4*/*TLR9* for inflamed VKC).

**Figure 2:**
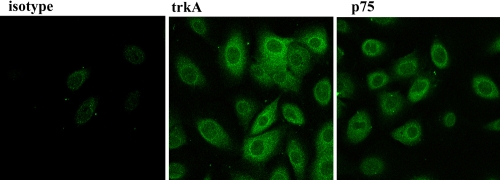
TrkA^NGFR^/p75^NTR^ protein expression in primary untouched VKC conjunctival epithelial cells (VKC-ECs). Confocal analysis specific for trkA^NGFR^ (middle) and p75^NTR^ (right) in VKC-ECs. Control-isotype signal (left) was used in channel series acquisitions, and identical acquisition settings were applied for all images (60× oil immersion).

Since NGF ability to modulate *TLR4*/*TLR9* expression was hypothesized, dose-response studies were performed with exogenous NGF over a 24-h exposure. To perform these studies, a preliminary check of trkA^NGFR^ and p75^NTR^ protein expression was carried out in passaged VKC-ECs (P_1_) by confocal analysis. As depicted in Figure 2, VKC ECs expressed both trkA^NGFR^/p75^NTR^, indicating VKC-ECs as well as healthy ECs are suitable for in vitro**exposure to exogenous NGF.

### Molecular expression of *TLR4*/*TLR9* in VKC conjunctival ECs as a function of NGF exposure

**Figure 3:**
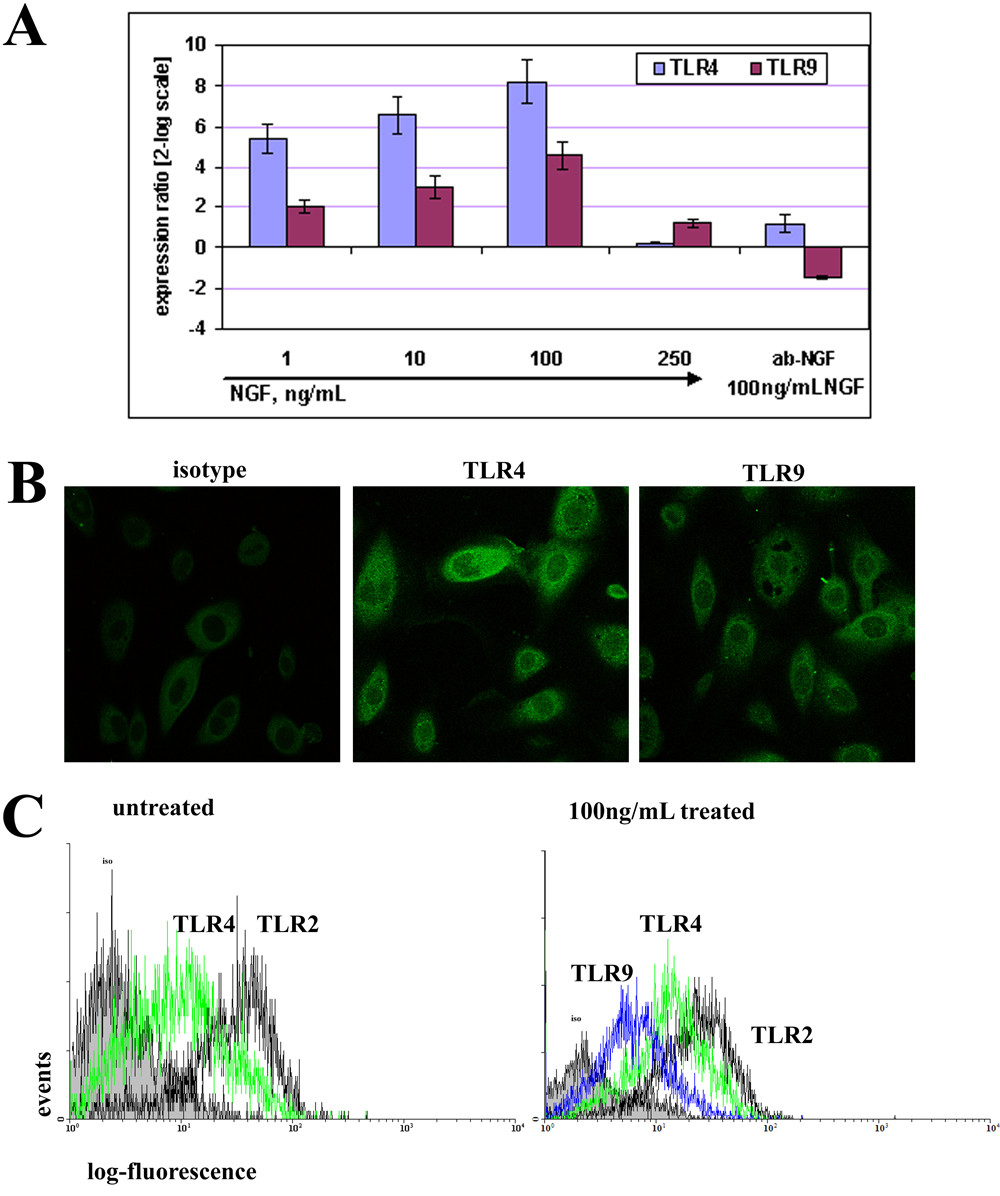
NGF modulates TLR4/TLR9 expression in VKC conjunctival epithelial cells (VKC-ECs). **A**: Relative *TLR4*/*TLR9* transcript expression in few passages VKC-ECs (P_1-2_). Short-term 1–250 ng/ml NGF exposure induced a steady *TLR4* and a slight *TLR9* increasing response (p<0.01, REST-ANOVA Tukey–Kramer-coupled analysis). As depicted, the maximum effect was observed at 100 ng/ml NGF, while its specific pretreatment with neutralizing anti-NGF antibody resulted in a downregulation of *TLR9* transcripts. Single Ct values were normalized to referring genes, and folds were calculated with respect to their untreated sister cells. **B**: Confocal analysis specific for TLR4 (middle) and TLR9 (right) in 100 ng/ml NGF-treated VKC-ECs. Control-isotype signal (left) was used in channel series acquisitions, and identical acquisition settings were done for all images (60× oil immersion). **C**: FACS showed a significant upregulation of both TLR4/TLR9 proteins (5,000 events, right). Note the absence of TLR9 signal in untreated VKC-ECs (left). TLR2 signal was used as internal control since no difference was observed in molecular analysis. Isotype-matched control antibody staining was in 10^1^ log decade (IF=7.65; solid area). The graphs are representative of three independent experiments for each sample, which gave identical results.

In parallel experiments for few passages VKC and healthy-control ECs, different *TLR4*/*TLR9* target gene responses were triggered as a function of short-term NGF. In VKC-ECs, the exposure to increasing NGF doses resulted in a rising modulation of *TLR4*/*TLR9* expression, with a consistent response for *TLR4* (5.40±0.74 at 1 ng/ml, 6.57±0.90 at 10 ng/ml, 9.07±1.17 at 100 ng/ml and 0.26±0.04 at 250 ng/ml NGF; p<0.01 REST–ANOVA Tukey–Kramer-coupled analysis) with respect to *TLR9* (2.02±0.33 at 1 ng/ml, 3.01±0.55 at 10 ng/ml, 5.55±0.77 at 100 ng/ml, and 1.23±0.17 at 250 ng/ml NGF; p<0.01 REST–ANOVA Tukey–Kramer-coupled analysis) when compared to untreated VKC-ECs (Figure 3A). This consistent *TLR4*/*TLR9* transcript upregulation was not monitored when VKC-ECs were incubated with NGF (100 ng/ml) in the presence of neutralizing anti-NGF antibodies (0.5 µg/ml; 1.21±0.39 for *TLR4* and -1.47±0.08 for *TLR9*, respectively). In few passages healthy-control ECs, *TLR4* expression was downregulated (2.97±0.51 at 1 ng/ml, -1.25±0.07 at 10 ng/ml, -4.18±0.19 at 100 ng/ml, and 0.16±0.05 at 250 ng/ml NGF; p<0.01 REST–ANOVA Tukey–Kramer-coupled analysis), while *TLR9* expression was upregulated (3.27±0.54 at 1 ng/ml, 1.12±0.24 at 10 ng/ml, 0.85±0.17 at 100 ng/ml, and 1.67±0.31 at 250 ng/ml NGF; p<0.01 REST–ANOVA Tukey–Kramer-coupled analysis), when compared to untreated ones. No significant difference in ΔCt occurred between primary (P_0_) and few passages (P_1-2_) VKC ECs. *TLR2* expression was used as an internal control.

### Biochemical evaluation of TLR4/TLR9 protein in VKC conjunctival ECs as a function of NGF exposure

Confocal analysis revealed that exposure to 100 ng/ml NGF increased intra-cytoplasm expression of TLR4/TLR9 and led to partial translocation at the cell surface (Figure 3B). Whole (surface and intracellular) TLR4/TLR9 staining in VKC-ECs was carried out by FACS analysis to verify whether TLR4/TLR9 transcript modulation resulted in an altered protein expression. As shown in Figure 3C, untreated VKC-ECs showed a consistent TLR4 expression in comparison to undetectable TLR9 (left), while a consistent TLR4/TLR9 expression was assessed after 100 ng/ml NGF exposure (right).

### Biochemical evaluation of cytokine release from VKC conjunctival ECs as a function of NGF exposure

Briefly, IFN-γ, IL-4, IL-10 and IL-12 p40 were quantified in conditioned media from passaged VKC-ECs either untreated or NGF treated. NGF did not influence IFN-γ, IL-12 p40, or IL-4 cytokine release in these VKC-ECs. Interestingly, NGF induced a significant decrease of IL-10 protein release in a dose-dependent fashion (% decrease: -5.67% at 1 ng/mL, -39.01% at 10 ng/ml, -59.23% at 100 ng/ml and -59.23% at 250 ng/ml NGF; p<0.01, ANOVA Tukey–Kramer post hoc) in comparison to untreated VKC-ECs (70.87±4.89 pg/ml).

## Discussion

This study confirmed and extended in vitro the previous observation of *TLR4*/*TLR9* expression in VKC conjunctival epithelium [20]. Short-term NGF exposure triggered a dose-response *TLR4*/*TLR9* transcript upregulation in cultured VKC-ECs, an effect confirmed by the related biochemical analysis. Finally, NGF exposure resulted in a decrease of IL-10 release without effects on IFN-γ, IL-4 or IL-12 p40, as detected by the analysis of the conditioned media.

On the ocular surface, NGF (via trkA^NGFR^/p75^NTR^) exerts pleiotrophic effects and contributes to the regulation of immune response and modulation of the tissue remodeling that occur in VKC [8–12,28]. Since high trkA^NGFR^ expression was detected in VKC conjunctival epithelium [11] and since NGF is impaired in VKC conjunctiva [11,12], we wondered whether exogenous NGF might contribute and/or modulate *TLR4*/*TLR9* expression in VKC conjunctival epithelium. Primary untouched cultures of VKC-ECs showed high *TLR4* and low *TLR9* expression, in line with those observed in VKC conjunctival epithelium [20] and conjunctival impression cytologies [unpublished data]. Focused NGF dose-response studies in few passages VKC-ECs resulted in a steady *TLR4* and a moderate *TLR9* upregulation at either molecular or biochemical levels. This NGF-mediated *TLR4*/*TLR9* upregulation appeared strictly associated with VKC phenotypes, as confirmed by the different NGF effects in healthy-control counterparts. The lower expressions of *TLR4* and *TLR9* at 250 ng/ml NGF strengthen suggest the contribution of both trkA^NGFR^/p75^NTR^ receptors in NGF-mediated *TLR4*/*TLR9* expression, according to kd binding [9].

The findings of this study suggest an additional NGF contribution in VKC inflammation. As the ocular surface expresses its own (resident) microflora and is constantly exposed to foreign substances, TLRs must be highly regulated to guarantee a quick response and prevent uncontrolled inflammatory launch [29,30]. As recently observed, TLRs are significantly affected and might contribute to the onset/ongoing of allergic and autoimmune manifestations [30]. Particularly, several data encompass the role of the Gram-negative ligand TLR4 in both acute and chronic Th1/Th2 disorders and more interesting their contribution in the homeostasis [14,15,30]. However, increasing evidence supports TLR9-based immunotherapy in allergic disorders, as shown in the prevention/treatment of experimental models of allergy [31,32]. In previous studies, the NGF/TLR4 association in experimental models and humans as well as the TLR9/p75^NTR^ association in cultured fibroblasts provide a possible link between NGF and innate immune response [21,22]. In this study, NGF-mediated *TLR4*/*TLR9* overexpression appears extremely attractive since it would imply an increased EC sensitization to microbial patterns. Alongside pathogens/commensal bacteria, which represent common TLR activator/modulator, other factors (growth factors, cytokines and neuropeptides) might contribute to the cell-surface expression of TLRs [19,33–36]. NGF-mediated *TLR4*/*TLR9* overexpression might favor a Th1 response according to the activated cytokine pattern [14,15]. In VKC tears and inflamed conjunctiva, several cytokines have been detected as a source of activated ECs [4,5]. Since NGF might be a product and might contribute directly or indirectly to the milieu of cytokines (IFN-γ/IL-4/IL-12 p40/IL-10) detected in VKC, NGF-treated VKC-ECs were also investigated in terms of cytokine release. As detected in the conditioned media, NGF did not influence the release of IFN-γ or IL-12 p40, while a decrease of IL-10 release was detected. This finding implies that NGF does not modulate Th1/Th2 balance via direct IFN-γ or IL-12 p40 release. The NGF-mediated IL-10 decrease might sustain *TLR9* overexpression since IL-10 neutralization significantly increased the deoxycytidylate-phosphate-deoxyguanylate (CpG)-induced IFN-α response in Peyer's patches [36,37].

An emerging paradigm indicates that commensal bacteria downregulate Th2 responses through nuclear factor-κB activation, while reduced exposure to microbial patterns might support a Th2 response (hygiene hypothesis) [17]. Therefore, a hypothetical regulation of microflora composition might offer the possibility to prevent and/or treat some Th2-linked diseases [18]. The recent observation that a viral infection might ameliorate both clinical signs and symptoms during active VKC as well as the observation that topical treatment with probiotics resulted in the resolution of VKC forms [38,39] support this hypothesis. In addition, NGF-mediated *TLR9* upregulation might be in line with the observation that synthetic immunostimulatory sequence (CpG motif ligands) activates *TLR9*, resulting in a more effective response than steroids in attenuating both asthma, allergic conjunctivitis and allergic rhinitis via specific Th2 downregulation [5,39,40]. On the other hand, NGF-mediated *TLR4* expression might suggest an NGF contribution in the regulation of bacterial clearance, as suggested by the observation that *TLR4* expression might drive a correct bacterial clearance in airway cells from children with airway bacterial colonization [41].

Cytokines, neuropeptides and growth factors might contribute to the modulation of *TLR* expression or be modulated by TLR-ligand activation. Recent information has noted the role of some soluble mediators (histamine), neurotransmitter (vasoactive intestinal peptide) or growth factors (epidermal growth factor receptor) in the modulation of *TLR* expression in different cell types [14,15,42,43]. According to the NGF pathway expression in VKC conjunctiva, exogenous NGF might contribute, either alone or in combination, to modulate innate response by counteracting *TLR9* downregulation observed in VKC epithelium. This NGF-mediated *TLR9* expression would allow a prompt response to specific exogenous and/or eventual synthetic ligands (probiotics/CpG-DNA). The possibility that other inflamed tissue-released factors might favor or counteract this NGF-mediated *TLR9* modulation should be taken into consideration.

Overall, the finding of this study support a theoretical NGF modulation of immune/adaptive response by means of *TLR4*/*TLR9* overexpression in VKC-ECs. The possible interaction between NGF and *TLR4*/*TLR9* in VKC-ECs could lead to a new and higher comprehension of VKC pathology and to the development of  nonconventional therapies [31,44].
